# Altered Gut Microbiota and Short-chain Fatty Acids in Chinese Children with Constipated Autism Spectrum Disorder

**DOI:** 10.1038/s41598-023-46566-2

**Published:** 2023-11-04

**Authors:** Jianquan He, Xiuhua Gong, Bing Hu, Lin Lin, Xiujuan Lin, Wenxiu Gong, Bangzhou Zhang, Man Cao, Yanzhi Xu, Rongmu Xia, Guohua Zheng, Shuijin Wu, Yuying Zhang

**Affiliations:** 1 College of Rehabilitation Medicine, Fujian University of Traditional Chinese Medicine, Fuzhou, China; 2 Department of Rehabilitation, School of Medicine, Zhongshan Hospital of Xiamen University, Xiamen University, Xiamen, China; 3 Xiamen Institute of Big Data of TCM Constitution and PreventiveTreatment for Disease, Xiamen, China; 4 School of Nursing, Qingdao University, Qingdao, China; 5 Department of Pediatrics, Yichun People’s Hospital, Yichun, China; 6 School of Medicine, Xiamen University, Xiamen, China; 7 Xiamen Treatgut Biotechnology Co., Ltd, Xiamen, China; 8 Clinical Research Institute, The Second Affiliated Hospital of Fujian University of Traditional Chinese Medicine, Fuzhou, China; 9 College of Nursing and Health Management, Shanghai University of Medicine and Health Sciences, Shanghai, China; 10 Xiamen Food and Drug Evaluation and Adverse Reaction Monitoring Center, Xiamen, China; 11 Department of Gastroenterology, Weifang People’s Hospital, Weifang, China

**Keywords:** Clinical microbiology, Autism spectrum disorders, Bacterial techniques and applications, Predictive medicine, Predictive markers, Digestive signs and symptoms

## Abstract

Gastrointestinal symptoms are more prevalent in children with autism spectrum disorder (ASD) than in typically developing (TD) children. Constipation is a significant gastrointestinal comorbidity of ASD, but the associations among constipated autism spectrum disorder (C-ASD), microbiota and short-chain fatty acids (SCFAs) are still debated. We enrolled 80 children, divided into the C-ASD group (n = 40) and the TD group (n = 40). In this study, an integrated 16S rRNA gene sequencing and gas chromatography–mass spectrometry-based metabolomics approach was applied to explore the association of the gut microbiota and SCFAs in C-ASD children in China. The community diversity estimated by the Observe, Chao1, and ACE indices was significantly lower in the C-ASD group than in the TD group. We observed that *Ruminococcaceae_UCG_002, Erysipelotrichaceae_UCG_003*, *Phascolarctobacterium*, *Megamonas*, *Ruminiclostridium_5*, *Parabacteroides*, *Prevotella_2*, *Fusobacterium*, and *Prevotella_9* were enriched in the C-ASD group, and *Anaerostipes*, *Lactobacillus*, *Ruminococcus_gnavus_group*, *Lachnospiraceae_NK4A136_group*, *Ralstonia*, *Eubacterium_eligens_group,* and *Ruminococcus_1* were enriched in the TD group. The propionate levels, which were higher in the C-ASD group, were negatively correlated with the abundance of *Lactobacillus* taxa, but were positively correlated with the severity of ASD symptoms. The random forest model, based on the 16 representative discriminant genera, achieved a high accuracy (AUC = 0.924). In conclusion, we found that C-ASD is related to altered gut microbiota and SCFAs, especially decreased abundance of *Lactobacillus* and excessive propionate in faeces, which provide new clues to understand C-ASD and biomarkers for the diagnosis and potential strategies for treatment of the disorder. This study was registered in the Chinese Clinical Trial Registry (www.chictr.org.cn; trial registration number ChiCTR2100052106; date of registration: October 17, 2021).

## Introduction

Autism spectrum disorder (ASD) is a category of complex neurodevelopmental disorders characterized by diminished social skills, communication impairments, and restricted interests or repetitive behaviours^1^. ASD affects over 5 million Americans, with a prevalence of approximately 2.3% in children, and the prevalence of ASD in boys is 4.2 times higher than that in girls^2^. The incidence of ASD in China has also increased in recent years. According to a study, the incidence of ASD in China increased by approximately 3.9% in 2018^3^.

Many children with ASD experience co-occurring conditions, such as gastrointestinal (GI) problems and picky eating^4,5^. A comprehensive meta-analysis showed that children with ASD were more than 4 times as likely to have GI disorders as children without ASD, with constipation, diarrhoea, and abdominal pain being the most common symptoms^6^. Constipation is an important GI comorbidity of ASD, and the incidence of constipation increases with greater social impairments and reduced language skills^4^. The median prevalence of ASD patients with one or more GI symptoms is 46.8%^7^. The relationship between internalizing symptoms and GI problems in youth with ASD is bidirectional^8^. Moreover, improvement in GI symptoms led to a decrease in the severity of social and emotional disorders in constipated autism spectrum disorder (C-ASD) children^9^.The bidirectional interaction between the gut microbiota and the brain is known as the “microbiota-gut-brain axis^10–13^”. Changes in the gut microbiota and metabolite composition have been found in both human ASD patients and animal models of ASD^14,15^. Children with ASD have an abnormal gut microbiota composition compared with typically developing (TD) children^16–20^, and studies have also found higher levels of certain pathogenic bacterial species in children with ASD than in normal children^21^. Clostridia, *Bacteroidetes*, and *Dorea* significantly increased in ASD, producing metabolites including short-chain fatty acids (SCFAs) such as propionate (PPA) and butyrate^19^. Previous studies have mainly focused on altered metabolites and gut microbiota of ASD patients^22,23^ or on altered gut microbiota of C-ASD^24^. A study by Wong et al. did not recruited girls^25^, which compared the microbiome of ASD with and without GI symptoms. However, few studies have focused on SCFAs in the faeces of C-ASD. A study by Alshammari et al. found that the highest incidence of *Clostridium perfringens*, a PPA-related bacteria, was found in the C-ASD group^26^. While numerous studies have highlighted differences in gut microbiota between individuals with ASD and TD, the search for beneficial bacteria or viable metabolic products to clinically treat ASD has been unsuccessful. This complex array of factors influencing gut microbial communities may stem partly from the interaction between culture, diet, environment, and customs constitutes. However, there is currently insufficient information available regarding the composition of gut microbiota and SCFAs in children with C-ASD in southern China.

In this study, we aimed to clarify the differences in faecal bacterial diversity and SCFAs levels in C-ASD children and investigate the association of the gut microbiota and SCFAs with C-ASD clinical parameters. Based on the representative discriminant model of dominant genera, we aimed to identify a universal set of microbial biomarkers to predict C-ASD.

## Results

### Characteristics and clinical indices of the participants

**Figure 1:**
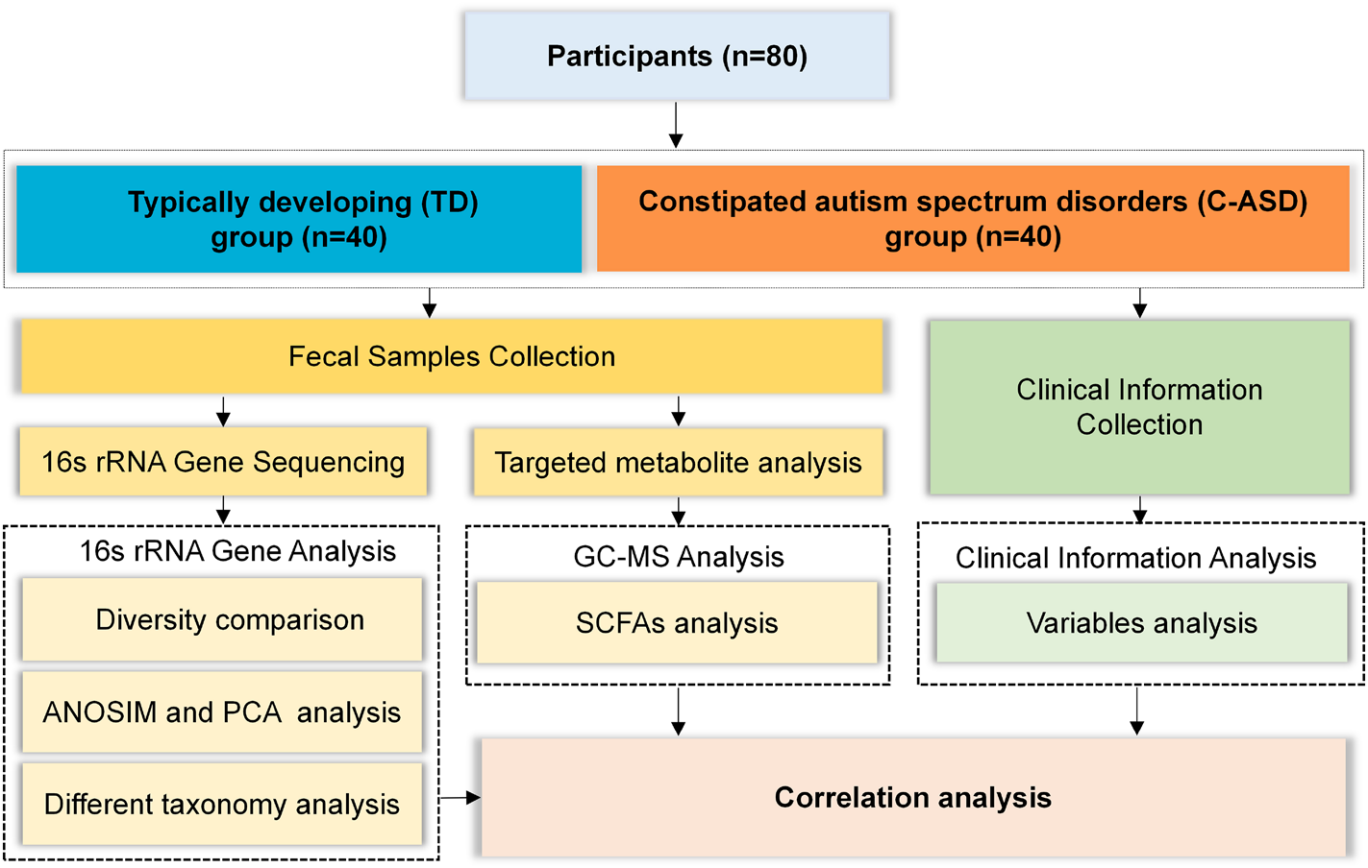
Flow diagram of this study.

After application of the inclusion and exclusion criteria, 40 TD children and 40 children with C-ASD were enrolled, and their data were analysed (Fig. 1). The patients’ clinical characteristics (including maternal age, gestational age, delivery mode, feeding patterns, maternal educational level, maternal smoking history, maternal drinking history, paternal educational level, paternal smoking history, and paternal drinking history) were compared between the TD and C-ASD groups. Additionally, we compared age, sex, height, weight, body mass index (BMI), birth weight, and Children’s Eating Behaviour Questionnaire (CEBQ) score between the TD group and the C-ASD group. There were no differences in all comparisons shown in Table 1. In the CEBQ scores, food fussiness, satiety responsiveness and desire to drink subscale scores were notably higher in the C-ASD group (Appendix 2. Table A1).

**Table 1:** Characteristics of study participants.

Variables	TD group	C-ASD group	F/X^2^	*P-value*
Sample size	40	40		
Age (year)	5.83 ± 1.28	5.30 ± 1.34	0.012	0.077
Sex (n, %)				
Male	27(67.50%)	30(75.00%)	0.549	0.459
Female	13(32.50%)	10(25.00%)		
Height (cm)	109.40 ± 7.47	106.90 ± 8.84	0.079	0.176
Weight (kg)	17.98 ± 2.431	16.84 ± 3.10	0.000	0.072
BMI (kg/m^2^)	14.96 ± 0.88	14.74 ± 1.55	8.232	0.154
Birth weight (kg)	3.30 ± 0.52	3.27 ± 0.56	0.561	0.790
Maternal age (year)	27.7 ± 3.81	28.03 ± 3.65	0.005	0.698
Gestational age (n, %)				
Term	39(97.50%)	36(90.00%)	1.920	0.166
Preterm	1(2.50%)	4(10.00%)		
Delivery mode (n, %)				
Natural birth	27(67.50%)	27(67.50%)	0.000	1.000
Cesarean section	13(32.50)	13(32.50)		
Feeding patterns (n, %)				
Breastfeeding	38(95.00%)	38(95.00%)	0.000	1.000
Artifificial feeding	2(5.00%)	2(5.00%)		
Maternal educational level (n, %)				
Primary or less	3(7.50%)	0(0.00%)	3.239	0.198
Secondary	8(20.00%)	10(25.00%)		
University or above	29(72.50%)	30(75.00%)		
Maternal smoking history (yes/no)	2/38	0/40	2.571	0.109
Maternal drinking history (yes/no)	6/34	3/37	1.127	0.288
Paternal educational level (n, %)				
Primary or less	1(2.50%)	2(5.00%)	0.373	0.830
Secondary	13(32.50%)	12(30.00%)		
University or above	26(65.00%)	26(65.00%)		
Paternal smoking history (yes/no)	13/27	12/28	0.058	0.809
Paternal drinking history (yes/no)	18/22	17/23	0.051	0.822
CEBQ	96.93 ± 13.24	99.68 ± 10.60	1.025	0.308
CARS	NA	36.80 ± 3.78	/	/
ABC	NA	67.48 ± 6.57	/	/

*TD* typically developing; *C-ASD* constipated autism spectrum disorders; *CEBQ* children’s eating behavior questionnaire; *CARS* childhood autism rating scale; *ABC* autism behavior checklist; *NA* not applicable.

The *p* value was compared between TD and C-ASD groups.

Data are expressed as mean ± standard deviation when applicable.

### Comparison of the diversity of the gut microbiota between the TD and C-ASD groups

**Figure 2:**
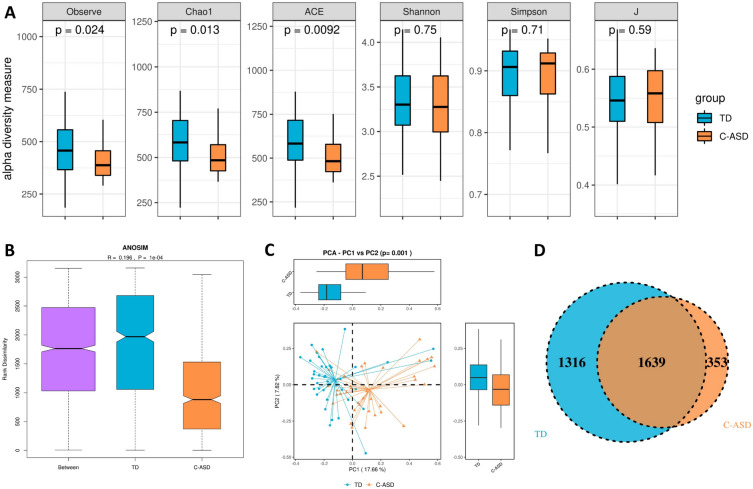
Decreased bacterial richness and diversity in the C-ASD group. (**A**) Comparison of bacterial alpha diversity indexes, including Observe, Chao1, ACE, Shannon, Simpson, and J. (**B**) ANOSIM was used to analyse the significant differences in colony distribution between the C-ASD group and the TD group (R = 0.0196, *p* = 1e-04). (**C**) PCoA revealing the bacterial communities between t the C-ASD group and the TD group on the PCoA1 vs PCoA2 axis (*p* = 0.001). (**D**) Venn diagram showing the differences in bacterial community structures between samples from the C-ASD group and the TD group.

The alpha diversity estimated by the Observe, Chao1, and ACE indices was significantly lower in the C-ASD group than in the TD group (*p* < 0.05). There was no statistically significant difference between the TD group and the C-ASD group in the J index, Simpson index, or Shannon index (*p* > 0.05) (Fig. 2A). Analysis of similarities (ANOSIM) was applied to compare within- and between-group similarity through a distance measure to evaluate the null hypothesis that the average grade resemblance between samples within a group aligned with the average rank similarity between samples belonging to different groups. There were marked differences in the population distribution between the TD group and the C-ASD group (Fig. 2B). PCoA was performed by using the distance matrix calculated from the species composition of the sample. The x axis and the y axis represent the contribution of the first principal component (PCoA1; 17.66%) and the second principal component (PCoA2; 7.82%), respectively (*p* = 0.001) (Fig. 2C). The Venn diagram indicates the shared and unique OTUs of the gut microbiota between the TD and C-ASD groups. The area of the circle indicates the quantity of OTUs. There were 1639 shared OTUs and 1316 unique OTUs in the TD group and 353 unique OTUs in the C-ASD group (Fig. 2D).

### Discriminative taxa between the TD and the C-ASD group

**Figure 3:**
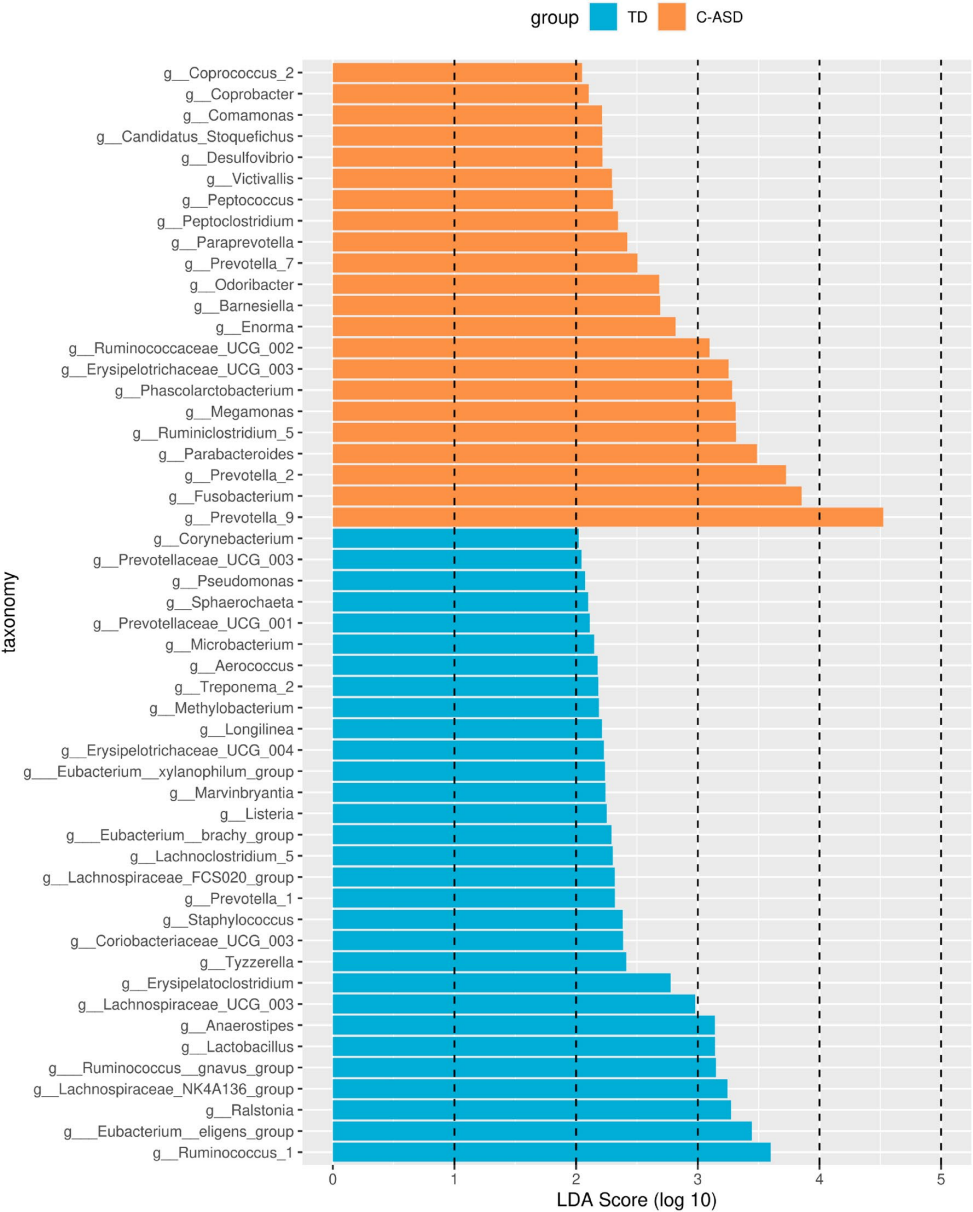
Discriminative taxa between the TD group and C-ASD group. LEfSe can be used to analyse the differences between groups and identify different microbial species, and this information can be used to develop biomarkers and promote other studies. In the above two syndrome types, the Linear discriminant analysis threshold was set as 3 to screen the characteristic flora of the corresponding syndrome types.

**Figure 4:**
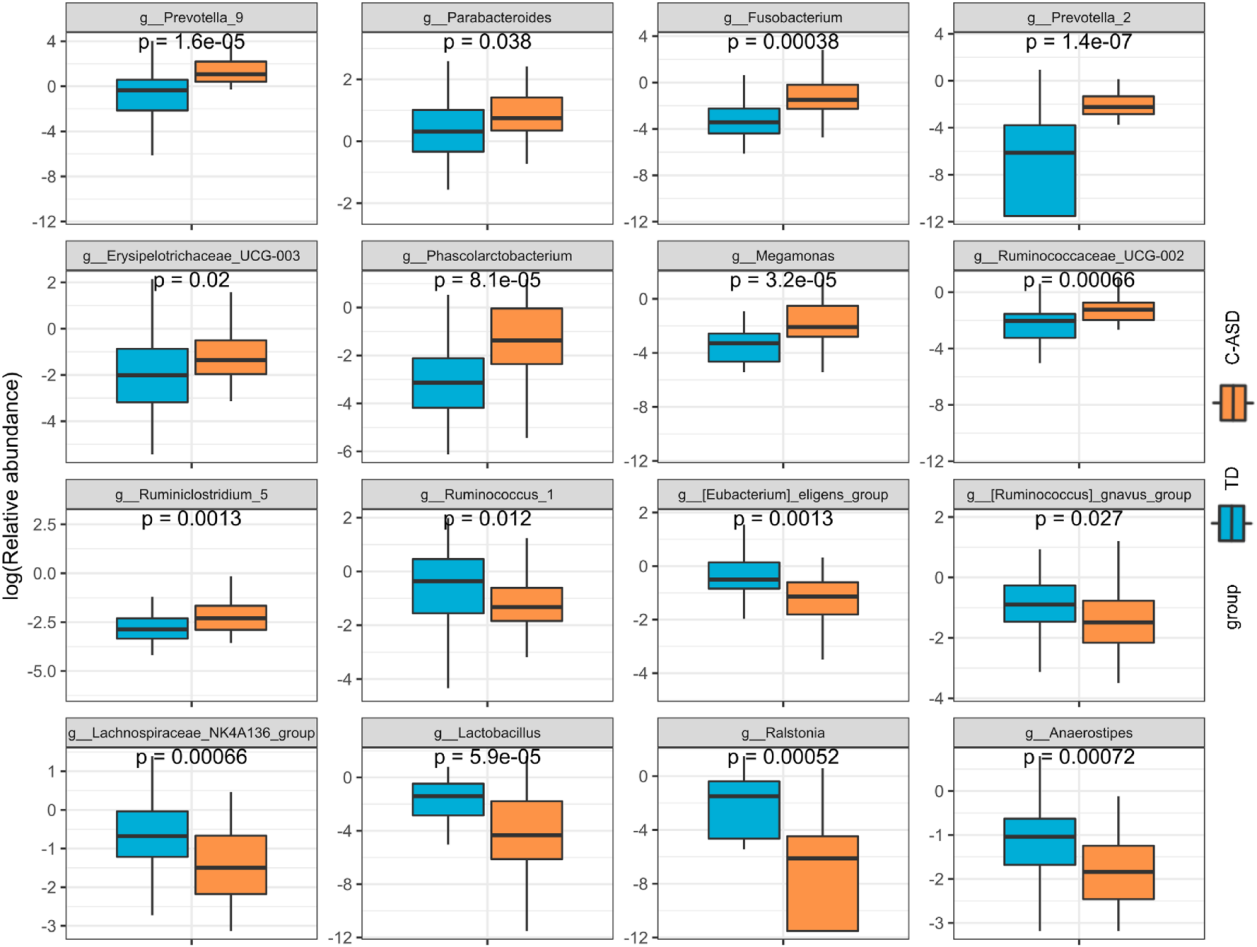
The relative abundance genus levels (LDA > 3, LEfSe) of gut microbiota between the TD group and the C-ASD group.

LEfSe can be used to analyse the differences between groups and identify differences in microbial species. In the TD group and the C-ASD group, the Linear discriminant analysis threshold was set at 3 to screen the 16 characteristic taxa of the corresponding groups. *Ruminococcaceae_UCG_002, Erysipelotrichaceae_UCG_003*, *Phascolarctobacterium*, *Megamonas*, *Ruminiclostridium_5*, *Parabacteroides*, *Prevotella_2*, *Fusobacterium*, and *Prevotella_9* were enriched in the C-ASD group, and *Anaerostipes*, *Lactobacillus*, *Ruminococcus_gnavus_group*, *Lachnospiraceae_NK4A136_group*, *Ralstonia*, *Eubacterium_eligens_group,* and *Ruminococcus_1* were abundant in the TD group (Fig. 3 and 4). The relative abundances of Solibacterales, *Corynebacterium*, *Brevibacterium*, etc., were significantly higher in the TD group than in the C-ASD group. Furthermore, the relative abundances of Acidobacteriales, *Enorma*, *Barnesiella*, etc., were significantly higher in the C-ASD group than in the TD group (Appendix 3: Figure A2). Furthermore, the 20 distinguished pathways with lower enrichment in the C-ASD group were identified to determine the potential interaction mode (Appendix 4: Figure A3).

### Comparison of SCFAs levels detected in faeces between the TD and the C-ASD group

**Figure 5:**
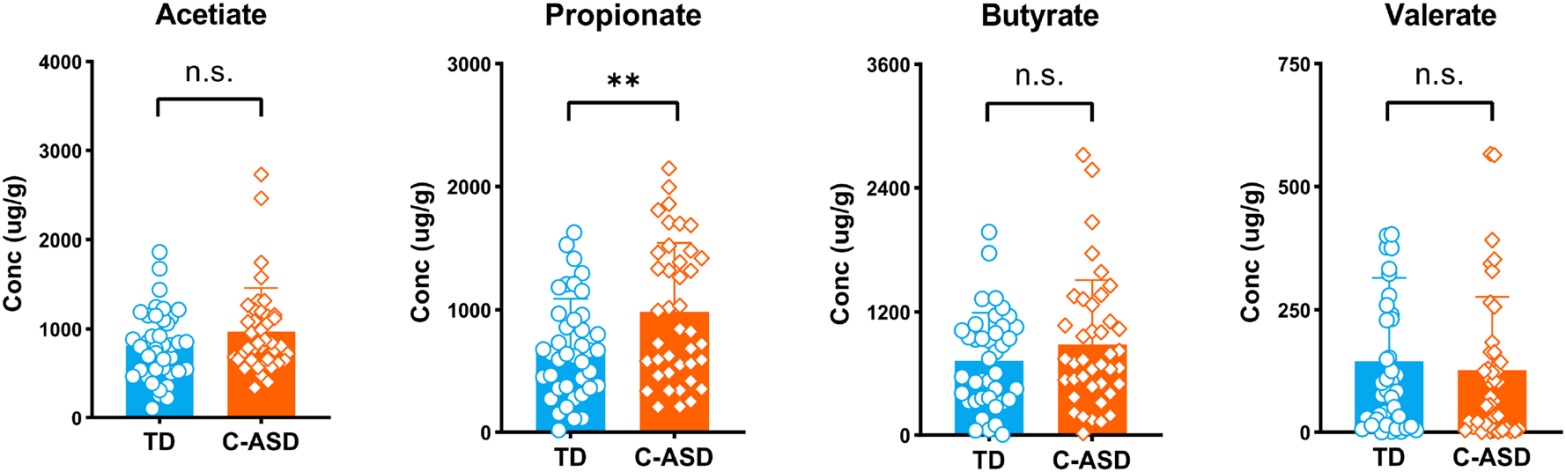
The levels of acetic, propionate, butyrate, and valerate acid in feces. Each value was presented as the mean ± SEM. *p* < 0.05.*.

We compared the SCFAs levels in the faeces of the TD group and the C-ASD group, including acetate, PPA, butyrate, valerate, isobutyrate, isovalerate, and hexanoate. The results showed that the faecal PPA levels in the C-ASD group were significantly higher than those in the TD group (*p* < 0.05), and there was no significant difference in acetate, butyrate, valerate, isobutyrate, isovalerate, and hexanoate levels between TD and C-ASD group (Fig. 5 and Appendix 5: Figure A4).

### Association among differential bacterial taxa, SCFAs levels and clinical manifestations

**Figure 6:**
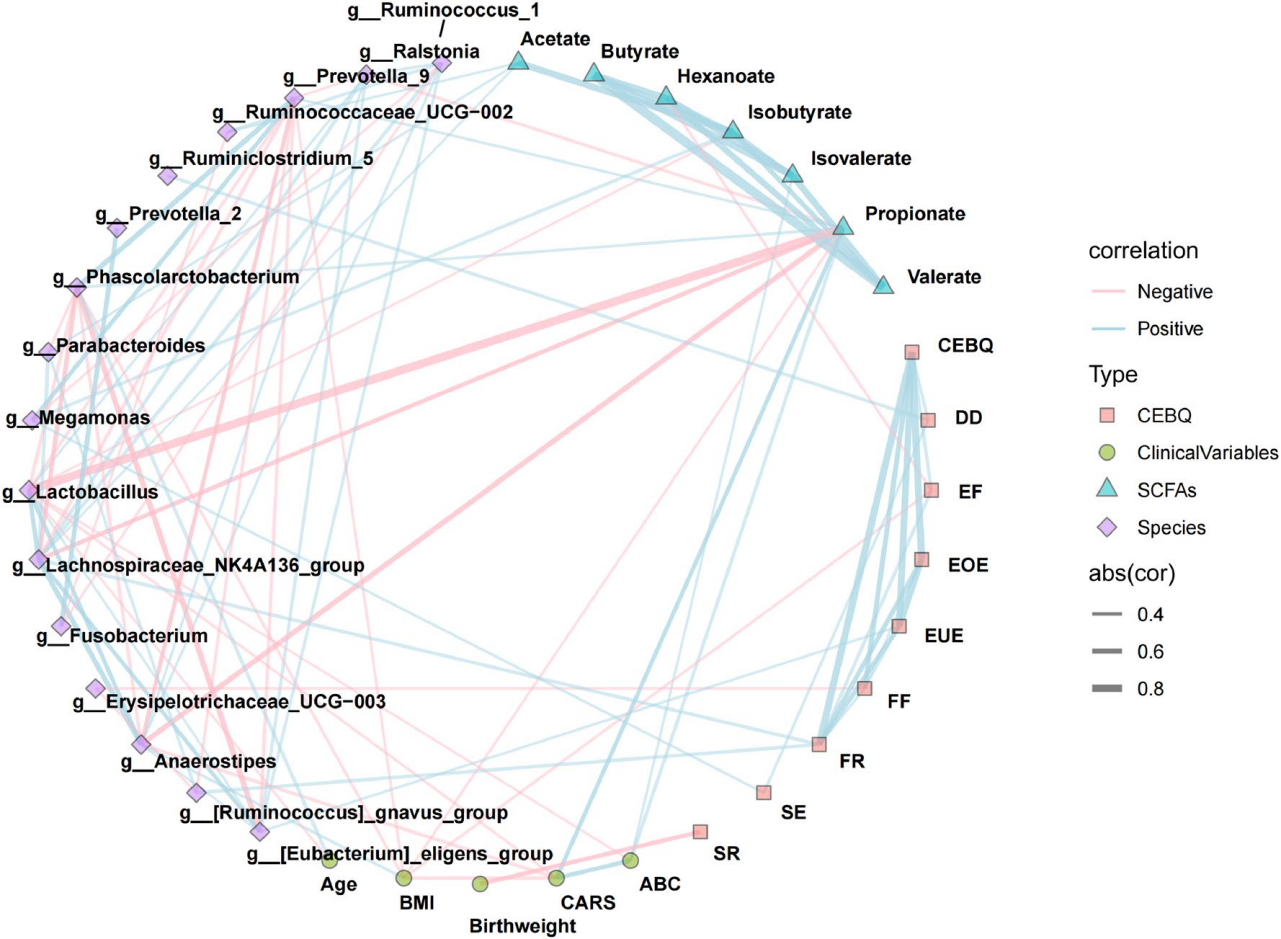
Integrative network among the discriminative genera, short-chain fatty acids and the clinical indexes. Network revealed significant (*P* < 0.05) between differentially abundant taxa or SCFAs metabolites and clinical indexes in TD (n = 40) and C-ASD (n = 40), respectively. Lines connecting nodes indicate positive (blue) or negative (red) correlations.

The associations among the differential bacterial taxa, SCFAs levels, and clinical manifestations were investigated with Spearman’s correlation analysis (Fig. 6 and Appendix 6: Table A2). Notably, the level of acetate was positively correlated with *Parabacteroides* (*r* = 0.340, *P* = 0.032), *Ruminococcaceae_UCG-002* (*r* = 0.328,* P* = 0.039)*, Lachnospiraceae_NK4A136_group* (*r* = 0.314, *P* = 0.049). PPA, a SCFA elevated in C-ASD group, was negatively associated with TD group enriched genera, such as *Lachnospiraceae_NK4A136_group* (*r* =  − 0.502, *P* = 0.001), *Anaerostipes* (*r* =  − 0.590, *P* = 6.06E − 05), *Ralstonia* (*r* =  − 0.392, *P* = 0.012) , while PPA was positively associated with *Prevotella_9* (*r* = 0.361, *P* = 0.022) , *Phascolarctobacterium* (*r* = 0.367,* P* = 0.020), Autism Behaviour Scale (ABC) (*r* = 0.430, *P* = 0.005) and Children's Autism Rating Scale (CARS) (*r* = 0.492, *P* = 0.001) respectively. In addition, isovalerate was positively associated with ABC (*r* = 0.335, *P* = 0.034). Insufficient *Lactobacillus* (observed in C-ASD group) was negatively correlated with increased PPA (*r* =  − 0.901, *P* = 2.21E − 15), isobutyrate (*r* =  − 0.317,* P* = 0.046), ABC (*r* =  − 0.319, *P* = 0.045) and CARS (*r* =  − 0.353, *P* = 0.025). *Anaerostipes*, the genus increased in TD group, was negatively correlated with CARS (*r* =  − 0.416, *P* = 0.008).

**Figure 7:**
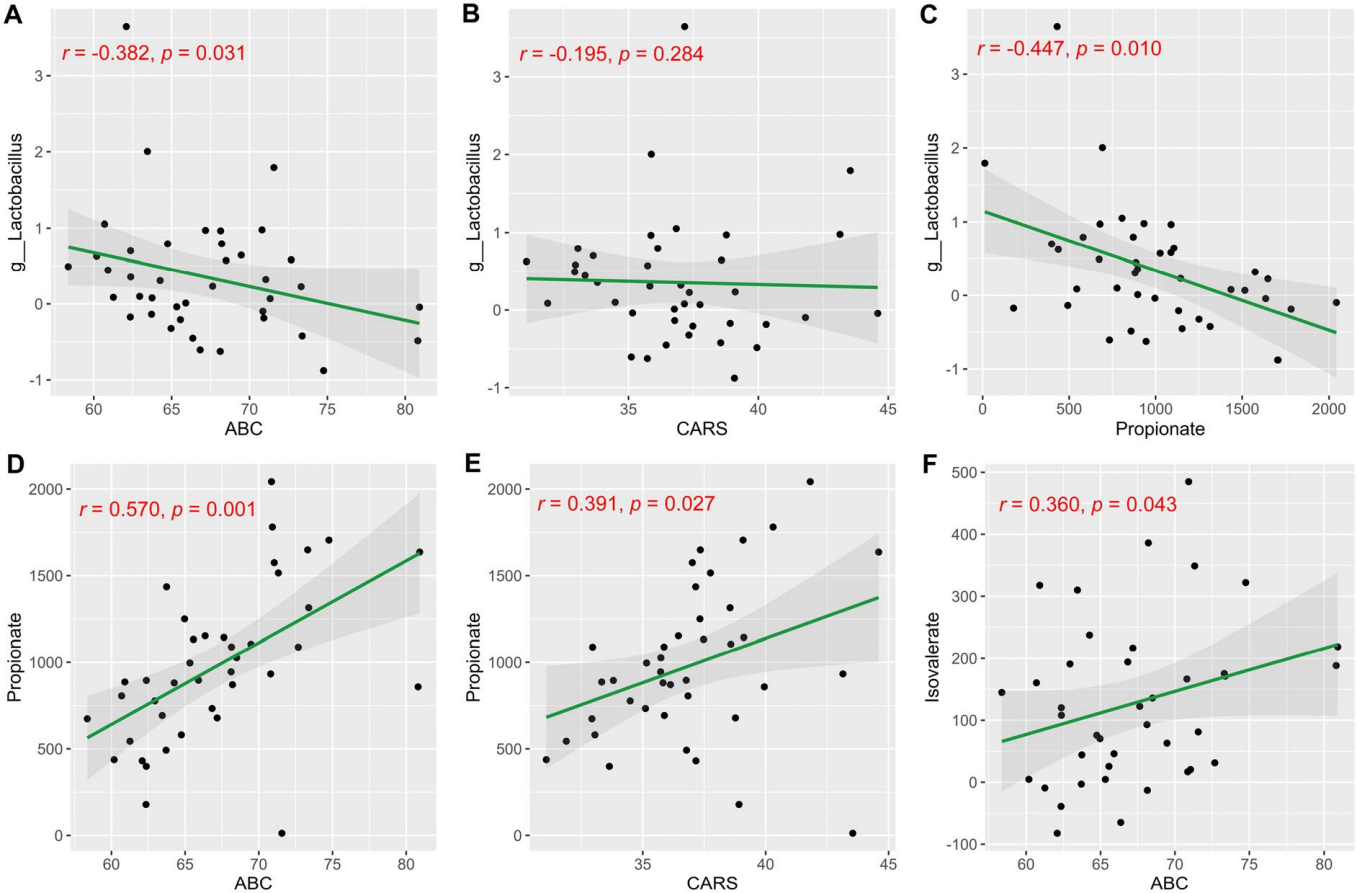
Relationship between differentially abundant correlative taxa or propionate and ASD scores (ABC and CARS) in C-ASD group (n = 40) by partial correlation analysis.

To further understand whether these disease-associated taxa and PPA contribute to disease severity, we tested for their correlations with clinical parameters using partial correlation analysis, controlling for age, BMI, birth weight, maternal age and CEBQ scores (including food fussiness, satiety responsiveness, and desire to drink). ABC score was negatively associated with *Lactobacillus* (*r* =  − 0.382, *P* = 0.031) (Fig. 7A). However, *Lactobacillus* has no association of CARS scores by partial correlation analysis (Fig. 7B). The PPA levels, which were higher in the C-ASD group, were negatively correlated with the abundance of *Lactobacillus* taxa (*r* =  − 0.447, *P* = 0.010) (Fig. 7C). We also found that PPA was positively correlated with ABC (*r* = 0.570, *P* = 0.001) and CARS (*r* = 0.391, *P* = 0.027) respectively (Fig. 7D,E), meanwhile isovalerate exhibited a tendency of positive association with ABC (*r* = 0.360, *P* = 0.043) (Fig. 7F). In conclusion, we found that PPA was positively correlated with the severity of ASD symptoms.

### Classification model

**Figure 8:**
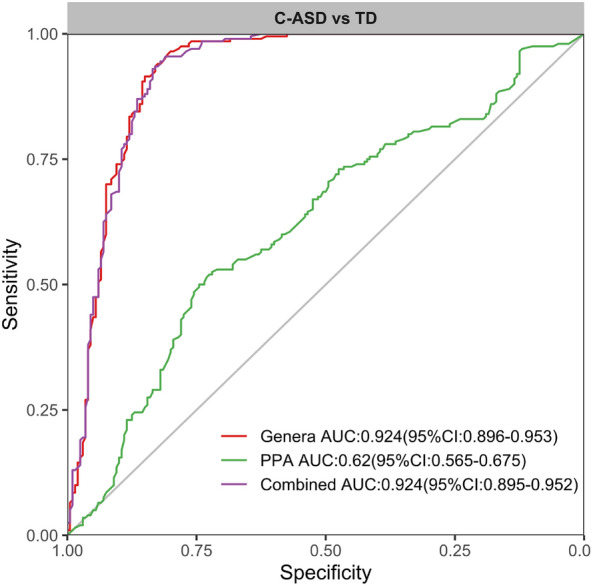
Receiver operating characteristic curve of training data based on selected features.

To investigate the potential utility of gut microbial and SCFAs profiles in C-ASD prediction, we built random forest models based on faecal taxonomic or SCFAs features to discriminate them. First, 16 candidate bacteria were selected according to the LEfSe analysis (LDA > 3). The discriminant model based on the 16 representative genera effectively distinguished the C-ASD group from the TD group (AUC = 0.924, 95% CI: 0.896–0.953) through fivefold cross-validation of the random forest model. However, the PPA-derived model did not improve the classification accuracy (AUC = 0.62, 95% CI: 0.565–0.675) (Fig. 8). Comparatively, the integration of PPA and 16 genera features showed similar accuracy in distinguishing C-ASD from TD (AUC = 0.924, 95% CI: 0.895–0.952) (Fig. 8). Taken together, these data indicated that the predictive models based on 16 key discriminatory bacterial taxa performed better than those based on PPA in discriminating C-ASD from TD.

## Discussion

This study investigated associations among the gut microbiota, SCFAs levels and C-ASD in Chinese children. Bacterial richness and diversity were decreased in Chinese children with C-ASD. Nine gut microbiota taxa were enriched in the C-ASD group, and seven gut microbiota taxa were enriched in the TD group. Faecal PPA levels were significantly higher in patients with C-ASD than in the TD group, and PPA levels were negatively correlated with *Lactobacillus* abundance*,* which in turn was negatively correlated with the severity of ASD.

Alterations of the gut microbiota in ASD have been described in previous reports. The abundance of *Lactobacillu*s, *Clostridia Cluster 1,* and *Desulfovibrio* was increased in children with ASD^20^. *Sutterella*, *Odoribacter* and *Butyricimonas* were more abundant in the ASD group; nevertheless, the abundance of *Veillonella* and *Streptococcus* were observably reduced^27^. In the genera level, there was a higher abundance of the genera *Ruminococcus, Bacteroides*, *Parabacteroides*, and *Phascolarctobacterium* in individuals with C-ASD^28,29^. However, in our research, we found that the abundances of *Ruminococcaceae_UCG_002, Erysipelotrichaceae_UCG_003*, *Phascolarctobacterium*, *Megamonas*, *Ruminiclostridium_5*, *Parabacteroides*, *Prevotella_2*, *Fusobacterium*, and *Prevotella_9* were higher in the C-ASD group. This inconsistency may be due to differences in genetic factors, disorder heterogeneity, dynamic microbiota development, locations, dietary habits, lifestyle, age, and detection methods.

The pathways involved in the microbiota-gut-brain axis in ASD include neuronal pathways, neural immune pathways and chemical signalling pathways^13^. The gut microbiota, such as *Lactobacillus rhamnosus JB1*^30^ and *L. reuteri*^31^, influence the brain via the vagal nerve pathway. Via chemical signalling pathways, the gut microbiota can also influence with the brain through microbial metabolites such as SCFAs, bile acids, and gamma aminobutyric acid^13^. The brains of mice colonized with ASD-associated microbiota displayed alternative splicing of ASD-relevant genes, and the gut microbiota adjust behaviours in mice through neuroactive metabolites (SCFAs), which contribute to the pathophysiology of ASD via the brain-gut axis^32^. In this study, there were no significant differences in acetate, butyrate, valerate, isobutyrate, isovalerate, and hexanoate levels between the TD group and the C-ASD group. We found that PPA levels were elevated in the C-ASD group compared with the TD group. In line with another study, the total amount of SCFAs was lower in ASD children, but PPA and acetate were found at higher levels in ASD children. In addition, the abundance of *Bacteroides* spp*.* was correlated with PPA levels in ASD individuals^33^. The association of PPA levels with the gut microbiota in the previous study was consistent with our study, in which PPA levels were negatively correlated with *Lactobacillus* abundance and positively correlated with *Prevotella_9, Phascolarctobacterium, Erysipelatoclostridium, Paraprevotella, Maihella,* and *Comamonas* abundance*.* In addition, *Alcaligenaceae* and *Porphyromonadaceae* abundance was also positively correlated with PPA levels^34^. In previous studies^35^, 111 strains of *Clostridium perfringens*, a PPA-related bacteria, have been isolated from faeces of children with ASD. Meanwhile, it has been demonstrated that Beta2 toxin encoding *cpb2* gene is associated with gastrointestinal diseases and significantly more common in the strains of *Clostridium perfringens*. As evidence indicates that *Clostridia* spp*.* produce PPA and worsen autistic symptoms, we inferred that the gut microbiota positively correlated with PPA levels may be potentially harmful microbiota in ASD. The survey on the isolation, identification and characterization of the PA-associated bacteria from the fecal samples of healthy and ASD children based on the traditional plate method was conducted by Yu et al.^36^. And, a PPA-resistant *L. plantarum strain 6–1* was successfully obtained.

Since the level of PPA was significantly higher in the C-ASD group and positively correlated with ABC and CARS scores in the current study, we hypothesized that PPA levels would be associated with the severity of ASD symptoms. Studies have demonstrated that rat models of ASD can be generated using various routes of PPA administration^37–39^. PPA-induced behavioural effects were demonstrated to coincide with ASD symptoms in patients. When PPA solution was injected into the ventricles of rat brains, the rats showed autism-like behaviour, and the structure of their nerve cells was altered^38,39^. PPA plays a significant role in ASD causation by altering several developmental molecular pathways, such as the PTEN/Akt, MAPK/ERK, mTOR/Gskβ, and cytokine-activated pathways, at the prenatal and neonatal stages^40,41^. A study showed that excessive PPA levels directly damaged neurons and was linked to neurological disorders^42^. From a physiological perspective, PPA can pass through the blood‒brain barrier and influence the central nervous system^39^. These results indicated that in C-ASD children, excessive PPA levels (as measured in faeces) might be an important factor aggravating ASD symptoms.

Regarding eating patterns, although no significant difference was found between the two groups in CEBQ scores, the C-ASD group showed more food fussiness, satiety responsiveness and desire to drink. A growing number of studies have established links between GI disorders and the gut microbiota in children with ASD. As a probiotic, *Lactobacillus* spp. can regulate the balance of intestinal microorganisms and help relieve diarrhoea and constipation^43^. *Lactobacillus* spp. also reduce the symptoms of constipation by shortening intestinal transit time, increasing the abundance of beneficial bacteria, and increasing faecal moisture^44–47^. Moreover, both animal and clinical studies have shown that probiotics can change the gut microbiota and thereby affect central nervous system function through the gut-brain axis. As a nondrug and relatively risk-free option, probiotics have gradually become an adjuvant treatment for children with ASD^48–50^. A study showed that after patients ate or were injected with a solution that contained *Lactobacillus* spp., the abundance of the gut microbiota changed significantly: the abundance of Firmicutes taxa, which produce PPA, was decreased, and the diversity of the gut microbiota was increased^51^. *Lactobacillus* spp. were shown to alleviate anxiety-like behaviours and increase dopamine levels in the prefrontal cortex in mice exposed to early life stress^52^. Probiotic treatment such as *Bifidobacteria* and *Lactobacilli* reduces the autistic-like excitation/inhibition imbalance in juvenile hamsters induced by orally administered propionic acid and clindamycin, mostly through increasing depleted γ-aminobutyric acid and Mg^2++^ and decreasing the excitatory neurotransmitter, glutamate^53^. Similarly, Aabed et al.^54^ found that probiotic/prebiotic treatments showed ameliorative effects on clindamycin and propionic acid-induced oxidative stress and altered gut microbiota in a rodent model of autism; however, *lactobacillus* had the strongest effect. In a four-week, randomized, double-blind, placebo-controlled study^55^, *Lactobacillus* spp. have also been shown to reduce the symptoms of ASD. Additionally, it has been shown that patients with ASD who take probiotics (including *Lactobacillus* spp.) have lower concentrations of PPA in the faeces^56^. These studies hint at the potential to administer probiotic interventions to reduce GI and ASD symptoms^57^. Therefore, a promising approach to enhance treatment efficacy in pediatric patients with C-ASD in the southern expanse of China may involve the application of a strategy utilizing PPA-resistant *Lactobacillus*.

Several scales, such as the CARS and ABC, are available to assess behaviours and symptoms associated with ASD. However, diagnosing ASD has always been difficult, and the condition is recognized at a late stage for treatment to have a major effect. Several studies have focused on biomarkers for determining ASD risk or assisting with diagnosis, such as proteomic analysis^58^, epigenome-wide analysis^59^, single-nucleotide polymorphism^60^, maackia amurensis lectin-II binding glycoproteins^61^, and the gut microbiota^62–65^. In our study, the use of microbial markers for the prediction of C-ASD was comprehensively assessed by 16 specific genera and PPA. The best-performing model achieved a high accuracy (AUC = 0.924) and included 16 important features that distinguished C-ASD from TD. Our results imply that the gut microbiome might provide new biomarkers to predict C-ASD.

There are some limitations of our study. First, we did not control for confounding factors of geographical differences, which can affect the gut microbiome. All included subjects were recruited from a city along the southeastern coast of China. Thus, the topography, climatic characteristics, and dietary patterns are similar. Second, intestinal bacteria were analysed based on 16S rRNA rather than metagenomics. Third, given the small sample size, more studies are needed to confirm these findings, as these data did not permit subgroup analysis. Since ASD is a highly heterogeneous disorder, it is more reasonable to perform a subgroup analysis to assess factors such as symptom severity. Finally, due to a cross-sectional study, the causal relationship among the gut microbiota, SCFAs levels and C-ASD remains unknown, but a complex association was observed.

## Conclusion

In summary, we identified different profiles of the gut microbiota and SCFAs in C-ASD and TD children. We investigated the associations among C-ASD clinical parameters, differential bacteria and PPA levels. These differences provide strong evidence that specific genera *Lactobacillus* within the gut represent a link between intestinal bacteria and C-ASD in children and could provide a theoretical basis for the treatment of C-ASD in the future.

## Materials and methods

### Human participants

Ethical approval was granted by the Ethics Committee of Zhongshan Hospital of Xiamen University (xmzsyyky (2021–171)) and registered in the Chinese Clinical Trial Registry (www.chictr.org.cn; trial registration number ChiCTR2100052106). All experiments were performed in accordance with relevant guidelines and regulations. Participants were recruited from Xiamen, Fujian Province, China, between November 1, 2021 and November 1, 2022. Inclusion criteria of the C-ASD group were as follows: (1) aged 3 to 10 years; (2) diagnosed with ASD according to the Diagnostic and Statistical Manual of Mental Disorders, fifth edition (DSM-V) criteria (CARS score ≥ 30 and ABC total score ≥ 67); and the International Statistical Classification of Diseases and Related Health Problems (ICD-10). (3) presence of GI comorbidities according to the Childhood Functional Gastrointestinal Disorders criteria (2016)^66^. (4) The guardians of each patient signed an informed consent form to allow the investigator to collect clinical data, faeces, and other samples. Two experienced child neuropsychiatrists determined the diagnosis of ASD. All parents were asked to complete the following questionnaires: CARS, ABC and CEBQ. Sex- and age-matched TD control children without GI disorders were included in the trial. The inclusion criteria for the TD children were as follows: (1) aged 3 to 10 years; and (2) in good health, without a clearly diagnosed disease. We adopted the following exclusion criteria before faecal sample collection: (1) definite diagnosis of Rett syndrome; (2) a clear history of brain injuries, cerebral palsy, encephalitis or other organic brain diseases; (3) a family history of serious mental illness or substance abuse; (4) use of probiotics, antibiotics, acid suppressors or other drugs affecting the gut microbiota in the last three months; (5) the presence of intestinal organic diseases, such as congenital megacolon, intestinal obstruction, intussusception and developmental disorder-related genetic diseases; (6) developmental quotient score < 35; or (7) participation in other clinical trials that would affect the evaluation of the study results. The confidentiality of participants results was maintained by de-identifying all samples. The participant's name was not used in all sections of the manuscript.

### Determination of clinical parameters

We collected basic information from all subjects (age, birth weight, height, and weight) and calculated the BMI using height and weight. We also interviewed the parents of the children to collect information on maternal age, gestational age, delivery mode, feeding patterns, maternal educational level, maternal smoking history, maternal drinking history, paternal educational level, paternal smoking history, and paternal drinking history. Children in the C-ASD group were assessed with the CARS and ABC. All children were evaluated with CEBQ.

### Sequencing and bioinformatics

Faecal samples were collected in sterile plastic cups and stored at − 80 °C for 1 h until further processing^67^. Faecal microbial DNA was extracted using a QIAamp DNA Stool Mini Kit (Qiagen, Hilden, Germany). PCR amplification was conducted using an ABI 2720 Thermal Cycler (Thermo Fisher Scientific, USA). We used Multiskan™ GO spectrophotometry (Thermo Fisher Scientific, USA) to quantify bacterial genomic DNA as the template for amplification of the V3-V4 hypervariable region of the 16S rRNA gene in three replicate reactions with forwards (Illumina adapter sequence 5’-CCTACGGGNBGCASCAG-3’) and reverse (Illumina adapter sequence 5’-GGACTACNVGGGTWTCTAAT-3’) primers. Replicate PCR products were pooled and purified with Agencourt AMPure XP magnetic beads (Beckman Coulter, USA). A TopTaq DNA Polymerase kit (TraTransgenichina) was used. The purity and concentration of sample DNA were assessed using a NanoDrop 2000 Spectrophotometer (Thermo Fisher Scientific, USA). Paired-end sequencing was performed by Treatgut Biotechnology Co., Ltd. with a HiSeq 2500 (Illumina, San Diego, CA, USA) with PE 250 bp reagents. After sequencing, raw paired-end reads were assembled using FLASH^68^ with the default parameters. Primers were removed using cutadapt, and clean tags were obtained by removing the lower reads using cutadapt^69^. To assign de novo operational taxonomic units (OTUs), we removed chimeric sequences and clustered sequences with 97% similarity using Research (V10.0.240)^70^. The representative sequences of OTUs were aligned to the SILVA132 database for taxonomic classification by RDP Classifier^71^ and aggregated to various taxonomic levels.

### SCFAs measurement

Frozen faecal samples were mixed with 1 mL of ice-cold saline, homogenized in a ball mill (4 min, 40 Hz), and then ultrasonically processed for 5 min in ice water, a process which was repeated three times. The supernatant was obtained by centrifugation (5,000 rpm, 20 min, 4 °C) and added to extracting solution containing 2-methylpentanoic acid (25 mg/L in methyl tert-butyl ether) as an internal standard. The mixture was then fully mixed by vortexing and oscillation and centrifuged for 15 min (10,000 rpm, 4 °C). After centrifugation, the supernatant was transferred into a fresh vial for gas chromatography‒mass spectrometry analysis. Gas chromatography‒mass spectrometry analysis was performed using an Agilent gas chromatograph coupled with an Agilent 5977B mass spectrometer. Helium was used as the carrier gas, and the initial temperature was set to 80 °C (1 min), subsequently increased to 200 °C (in 5 min), and then maintained (1 min) at 240 °C. The injection and ion source temperatures were 240 and 230 °C, respectively. The electron impact energy was -70 Eva. The mass spectrometry data were acquired in Scan/SIM mode.

### Statistical analyses and visualization

The rarefaction curves constructed from the sequenced data were basically stable, indicating that the sequenced data were basically stable at this sequencing depth (Appendix 1: Figure A1). The alpha diversity indices (Shannon, Simpson, ACE, and Chao1 indices) and bacterial richness (observed OTUs) and evenness (J) were calculated based on the OTU tables of the study. Detection of significant differences between the TD and C-ASD groups was performed with the Wilcoxon test. Differences in community structure across samples (beta diversity) were visualized by principal coordinates analysis (PCoA) plots based on Bray‒Curtis distance. Significance tests were determined using permutational multivariate analysis of variance (PERMANOVA) with 999 permutations in vegan^72^. Linear discriminant analysis effect size (LEfSe)^73^ was performed to identify taxa with differential abundance between the TD and C-ASD groups. We further explored the correlations among clinical data, abundance of different genera (LEfSe, LDA > 3) and faecal SCFAs by Spearman’s correlation analyses. Partial correlation analysis (PResiduals package) was used to evaluate associations between differentially abundant correlative taxa (*P* < 0.05, Spearman’s correlation analyses) or SCFAs and ASD scores (ABC and CARS) in C-ASD group. To evaluate functional differences in the gut microbiomes of the TD versus the C-ASD groups, we used PICRUSt^74^ to calculate the microbial abundances, assigned metabolic pathways to the gut microbiomes using KEGG and COG analyses, and then tested the differences between the two groups. To train multivariable statistical models for the prediction of TD and C-ASD groups, two levels of bacterial and SCFAs features were combined to develop prediction models using the random Forest R package v4.6–14. Finally, all predictions were used to calculate the area under the receiver operating characteristics curve (AUC) using the pROC R package v1.17.01. All statistical and correlational analyses were conducted in R (v3.6.0)^75^. Figures were plotted mainly using ggplot2 (v3.0.0)^76^.

### Ethical approval

This study was approved by the Ethics Committee of Zhongshan Hospital of Xiamen University (xmzsyyky (2021–171)) and registered in the Chinese Clinical Trial Registry (www.chictr.org.cn; trial registration number ChiCTR2100052106). All participants and/or their parents provided written informed consent for their participation in the study.

### Supplementary Information

Supplementary Information 1.

Supplementary Information 2.

Supplementary Information 3.

Supplementary Information 4.

Supplementary Information 5.

Supplementary Information 6.

## Data Availability

The datasets generated during and analysed during the current study are available from the corresponding author upon reasonable request. All sequence data is available from NCBIs Sequence Read Archive (SRA), Bio Project ID: PRJNA932561.
