# Supplementary material for: Altered Gut Microbiota and Short-chain Fatty Acids in Chinese Children with Constipated Autism Spectrum Disorder
Source: Sci Rep. 2023 Nov 4;13:19103. doi: 10.1038/s41598-023-46566-2 (PMC10625580; doi:10.1038/s41598-023-46566-2)
Supplement: Supplementary file 1 — Supplementary Information 1. [file 41598_2023_46566_MOESM1_ESM.docx]

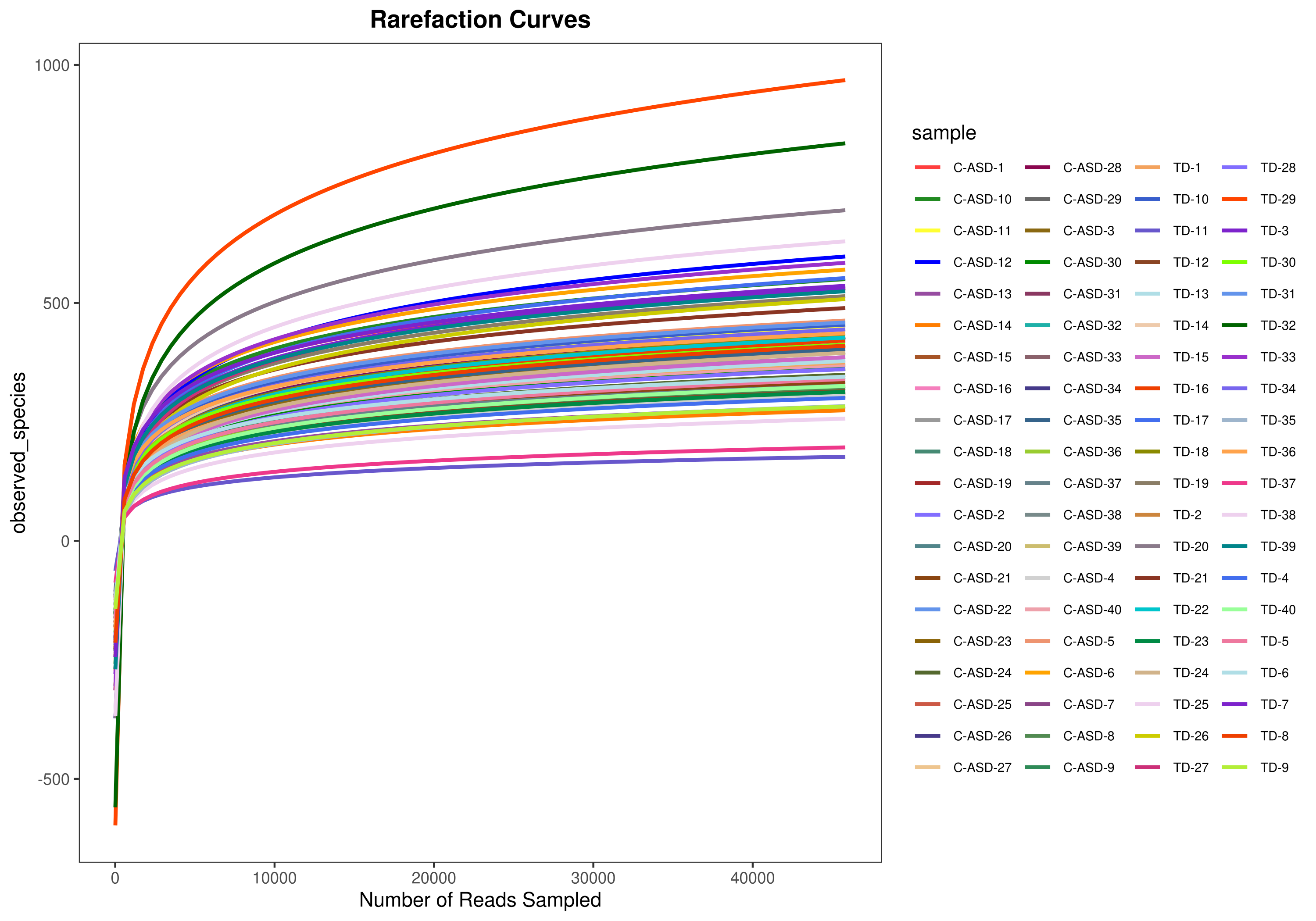


## Figure A1 Rarefaction curves in observed_species of all the samples.

The rarefaction curves constructed from the sequenced data has been basically stable, indicating that the sequenced data has been basically stable at this sequencing depth.
