# Supplementary material for: Altered Gut Microbiota and Short-chain Fatty Acids in Chinese Children with Constipated Autism Spectrum Disorder
Source: Sci Rep. 2023 Nov 4;13:19103. doi: 10.1038/s41598-023-46566-2 (PMC10625580; doi:10.1038/s41598-023-46566-2)
Supplement: Supplementary file 2 — Supplementary Information 2. [file 41598_2023_46566_MOESM2_ESM.docx]

| Table A1. Values of the Children's Eating Behavior Questionnaire. | | | | |
| --- | --- | --- | --- | --- |
|  | TD group | C-ASD group | *t-value* | *P-value* |
| EF: Enjoyment of food | 12.925±2.390 | 12.750±2.499 | -0.320 | 0.750 |
| EOE: Emotional overeating | 9.375±2.250 | 8.625±2.628 | -1.371 | 0.174 |
| DD: Desire to drink | 7.200±1.698 | 8.350±1.369 | 3.335 | 0.001 |
| FF: Food fussiness | 16.850±2.637 | 18.000±1.961 | 0.123 | 0.030 |
| EUE: Emotional undereating | 11.275±2.428 | 10.975±2.423 | -0.553 | 0.582 |
| FR: Food responsiveness | 14.025±3.309 | 13.075±2.912 | -1.363 | 0.177 |
| SR: Satiety responsiveness | 16.700±3.322 | 19.475±2.112 | 4.458 | ＜0.001 |
| SE: Slowness in eating | 8.575±1.583 | 8.425±1.394 | -0.450 | 0.654 |
