# Supplementary material for: Altered Gut Microbiota and Short-chain Fatty Acids in Chinese Children with Constipated Autism Spectrum Disorder
Source: Sci Rep. 2023 Nov 4;13:19103. doi: 10.1038/s41598-023-46566-2 (PMC10625580; doi:10.1038/s41598-023-46566-2)
Supplement: Supplementary file 3 — Supplementary Information 3. [file 41598_2023_46566_MOESM3_ESM.docx]

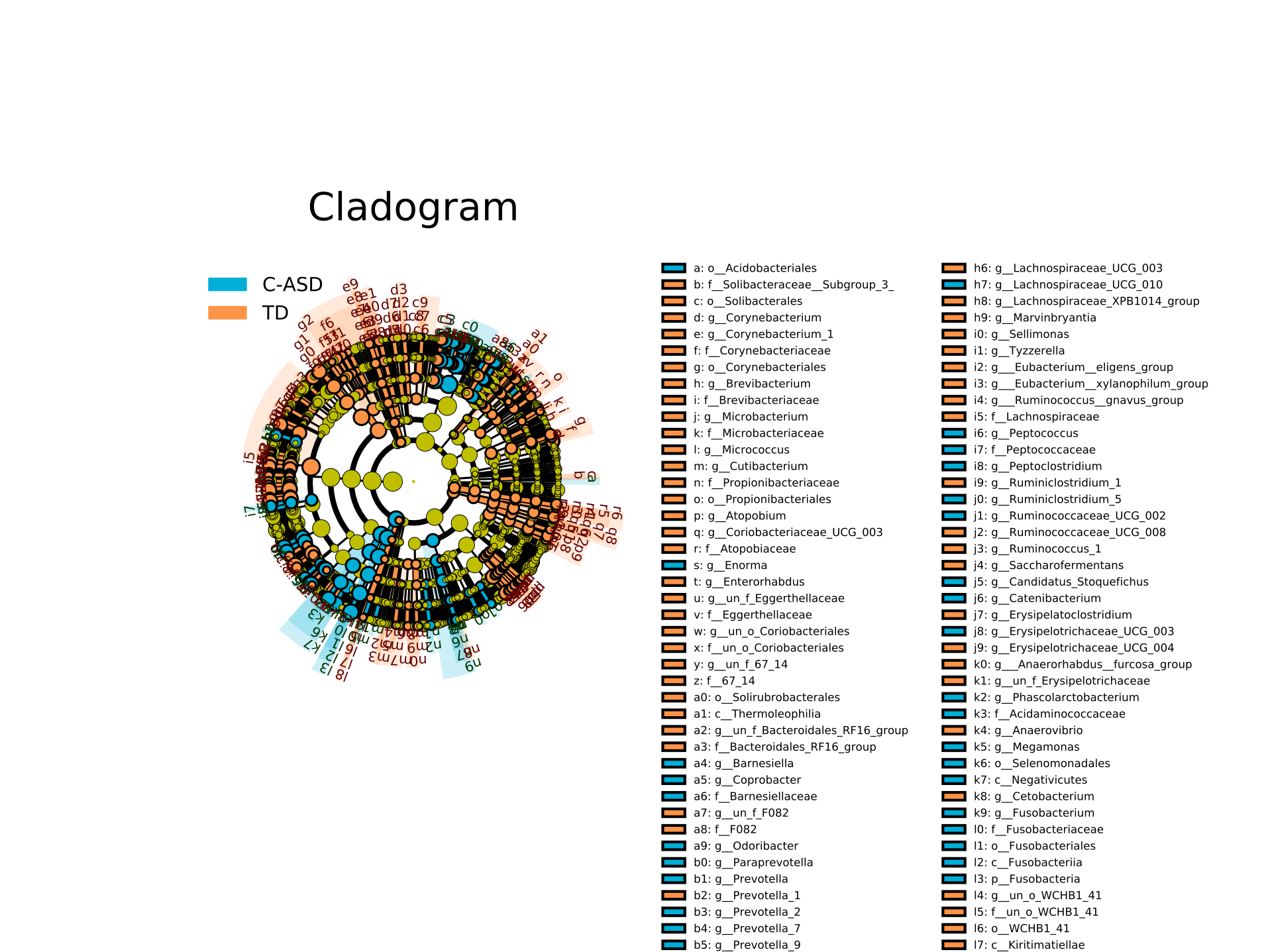


## Figure A2 The taxonomic representation of statistically and biologically differences between the TD group and the C-ASD group.

The colour of discriminative taxa represents the taxa is more abundant in the corresponding group (the C-ASD group in blue, the TD group in orange).
