# Supplementary material for: Altered Gut Microbiota and Short-chain Fatty Acids in Chinese Children with Constipated Autism Spectrum Disorder
Source: Sci Rep. 2023 Nov 4;13:19103. doi: 10.1038/s41598-023-46566-2 (PMC10625580; doi:10.1038/s41598-023-46566-2)
Supplement: Supplementary file 4 — Supplementary Information 4. [file 41598_2023_46566_MOESM4_ESM.docx]

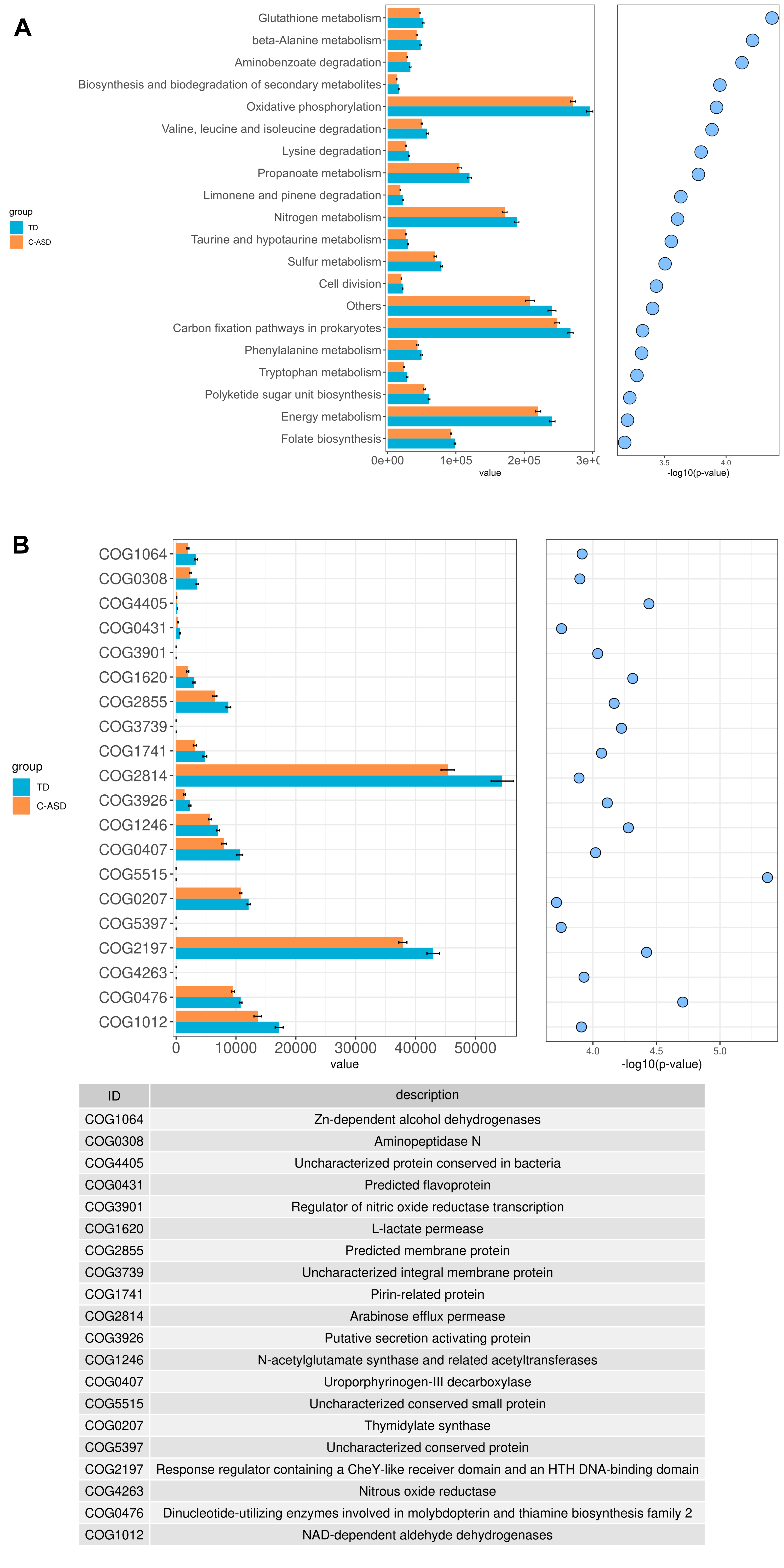


**Figure A3 Functional PICRUSt analysis among the TD group and the C-ASD group.** Kyoto Encyclopedia of Genes **(A)** and Genomes and cluster of orthologous groups **(B)** were used to further investigate the mechanism of intestinal flora.
