# Supplementary figures and images for: Altered Gut Microbiota and Short-chain Fatty Acids in Chinese Children with Constipated Autism Spectrum Disorder
Source: Sci Rep. 2023 Nov 4;13:19103. doi: 10.1038/s41598-023-46566-2 (PMC10625580; doi:10.1038/s41598-023-46566-2)

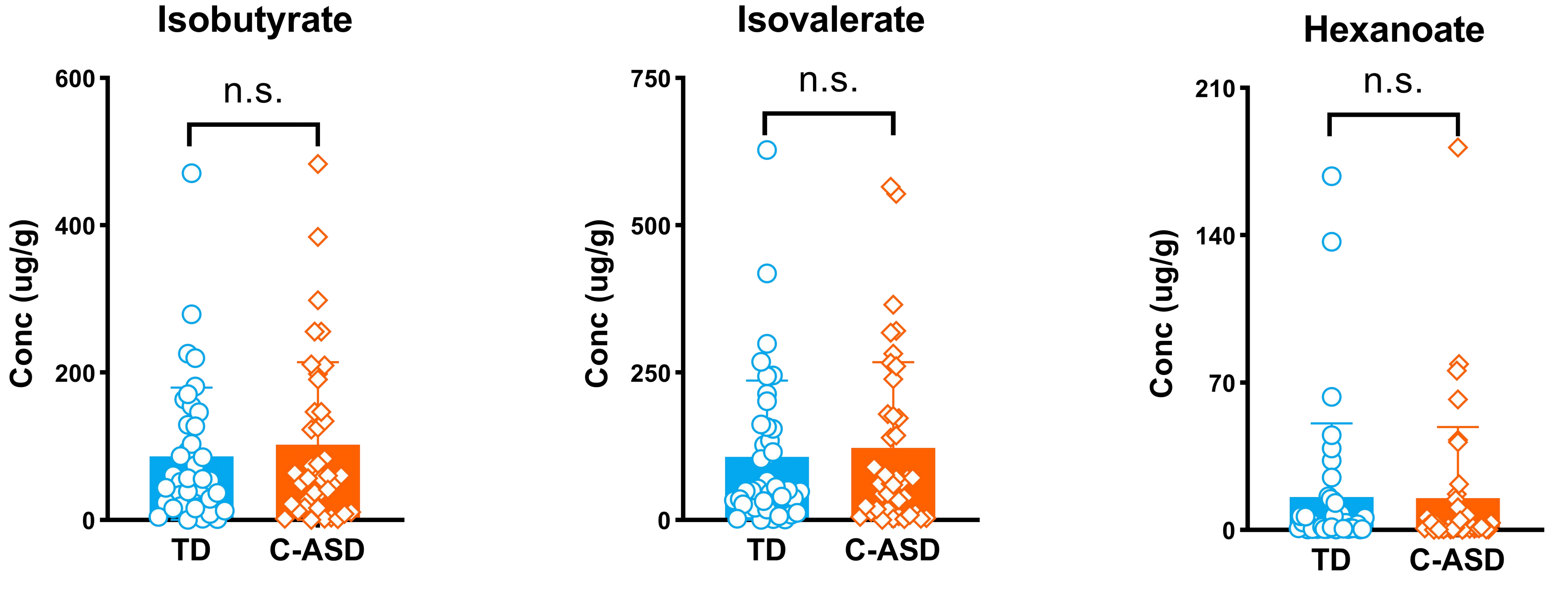


## Figure A4 The levels of isobutyrate, isovalerate, and hexanoate in feces.

Supplement: Supplementary file 5 — Supplementary Information 5. [file 41598_2023_46566_MOESM5_ESM.docx]
